# Lipid metabolic profiling and diagnostic model development for hyperlipidemic acute pancreatitis

**DOI:** 10.3389/fphys.2024.1457349

**Published:** 2024-10-24

**Authors:** Dongmei Ren, Yong Li, Guangnian Zhang, Tiantian Li, Zhenglong Liu

**Affiliations:** ^1^ Department of Hepatobiliary Surgery II, Affiliated Hospital of North Sichuan Medical College, Nanchong, China; ^2^ School of Basic Medical Sciences and Forensic Medicine, North Sichuan Medical College, Nanchong, China

**Keywords:** hyperlipidemic acute pancreatitis, lipidomics, metabolic biomarkers, diagnostic model, biomarkers

## Abstract

**Introduction:**

Hyperlipidemic acute pancreatitis (HLAP) is a form of pancreatitis induced by hyperlipidemia, posing significant diagnostic challenges due to its complex lipid metabolism disturbances.

**Methods:**

This study compared the serum lipid profiles of HLAP patients with those of a healthy cohort using ultra-performance liquid chromatography-tandem mass spectrometry (UPLC-MS/MS). Orthogonal partial least squares discriminant analysis (OPLS-DA) was applied to identify distinct lipid metabolites. Logistic regression and LASSO regression were used to develop a diagnostic model based on the lipid molecules identified.

**Results:**

A total of 393 distinct lipid metabolites were detected, impacting critical pathways such as fatty acid, sphingolipid, and glycerophospholipid metabolism. Five specific lipid molecules were selected to construct a diagnostic model, which achieved an area under the curve (AUC) of 1 in the receiver operating characteristic (ROC) analysis, indicating outstanding diagnostic accuracy.

**Discussion:**

These findings highlight the importance of lipid metabolism disturbances in HLAP. The identified lipid molecules could serve as valuable biomarkers for HLAP diagnosis, offering potential for more accurate and early detection.

## Highlights

1. This study reports the first-ever exploration of the metabolic characteristics of hyperlipidemic acute pancreatitis using a lipidomics platform.
2. Three hundred ninety-three differential metabolites were identified, distinguishing patients with hyperlipidemia-induced acute pancreatitis (HLAP) from healthy individuals.
3. The study identifies lipid metabolism, fatty acid metabolism, and glycerophospholipid metabolism as key pathways compromised in HLAP.
4. The lipid molecular diagnostic model demonstrates perfect predictive performance (area under the ROC curve = 1).
5. This research provides new theoretical foundations and molecular targets for diagnosing and treating hyperlipidemia-induced acute pancreatitis.

## Introduction

With the westernization of people’s lifestyles and changes in dietary structure, hyperlipidemia-induced acute pancreatitis (HLAP) has become the second leading cause of acute pancreatitis (AP) in China. Compared to other types of AP, patients with HLAP have a higher tendency for recurrence and a greater risk of progressing to severe pancreatitis (Zou et al., 2005). The mechanisms by which high triglyceride levels contribute to the onset and exacerbation of AP remain unclear. It has been observed clinically that only a portion of patients with hyperlipidemia progress to HLAP. Studies indicate that even though Types I, IV, and V hyperlipidemia primarily involve elevated triglycerides (TG), patients with Type I hyperchylomicronemia are more likely to develop HLAP (Gotoda et al., 2012; Tada et al., 2015). The lipid classification system proposed by the National Institutes of Health in the U.S. categorizes lipids into eight classes that regulate various life processes (Fahy et al., 2009; Zhi et al., 2021; Zhang et al., 2019). However, due to limitations in detection throughput, current research on the lipid metabolism of HLAP primarily focuses on serum total TG and free fatty acids (FFA) associated with damage to pancreatic acinar cells. HLAP patients exhibit significant disturbances in lipid metabolism, yet there is a lack of research characterizing the specific lipid metabolic abnormalities of HLAP. Therefore, a comprehensive understanding of the lipid metabolic characteristics of HLAP may offer new insights into the disease.

Metabolomics involves the quantitative analysis of all metabolites in a biological organism, aiming to identify specific and sensitive diagnostic or predictive metabolic markers while elucidating the connections between metabolites and physiological or pathological changes, thus potentially uncovering new therapeutic targets for diseases (Luo et al., 2018). Nuclear magnetic resonance (NMR) spectroscopy, liquid chromatography-mass spectrometry (LC-MS), and gas chromatography-mass spectrometry (GC-MS) are valuable tools for metabolomics or lipidomics analysis, each with its strengths and limitations (Kimhofer et al., 2015; Liang et al., 2016). LC-MS, combining the high separation capability of chromatography with the selectivity and sensitivity of mass spectrometry, is the most commonly used tool for detecting a broad range of endogenous metabolites in tissue or body fluid samples (blood, urine, tissue fluid), and it has been widely adopted in numerous studies (Deng et al., 2021). This study collected serum samples from HLAP patients and healthy controls, utilized LC-MS lipidomics technology to analyze the blood samples, compared metabolites, and identified differential metabolites that are meaningful for clinical diagnosis and mechanistic exploration.

## Materials and methods

### Sample source

The HLAP patients included in this study were recruited from the departments of Gastroenterology and General Surgery at the affiliated hospital of North Sichuan Medical College. Blood samples and clinical data were collected from patients upon admission. The collection period ranged from January 2020 to October 2020. The study was divided into two groups: the HLAP group (n = 24) and the healthy control group (Con, n = 24). Participants were randomly selected from eligible patients using a random number method, and the control group was matched by age and gender to minimize selection bias. The diagnosis criteria for HLAP patients referred to the 2012 revised Atlanta classification international consensus (Banks et al., 2013). Based on clinical severity, participants were divided into a mild group (n = 18, no organ failure, local or systemic complications) and a moderately severe group (n = 6, with transient organ failure, local complications, or exacerbation of comorbidities). Participants voluntarily consented to take part in the study, agreed to provide sufficient blood samples and access to complete medical records, and signed informed consent forms. Exclusion criteria comprised severe hepatic or renal diseases, current or prior malignancies, chronic kidney disease, and other conditions that could lead to elevated amylase levels or pancreatitis. Healthy controls were individuals undergoing routine health check-ups at the Health Examination Center of North Sichuan Medical College Hospital. Inclusion criteria for participants in the control group: (1) No restrictions on gender, with an age range of 18 to 85 years; (2) No significant symptoms, and examination and laboratory test results within normal ranges; (3) Participants were informed and agreed to participate in the study, and signed the informed consent form; (4) Participants were able to provide an adequate blood sample.

### Primary reagents

The methanol, methyl tert-butyl ether (MTBE), acetonitrile, isopropanol, formic acid, and ammonium formate used in the study were all of chromatographic grade and purchased from Fisher Scientific in the United States.

### Sample collection

Patients were selected based on inclusion and exclusion criteria, their medical histories were collected. Included patients were required to fast for at least 8 h prior to sample collection. In the morning, on an empty stomach, 5 mL of venous blood was collected and placed in ethylenediaminetetraacetic acid disodium salt (EDTA) vacuum blood collection tubes.

### Sample processing

After sample collection, samples were left to stand on an ice pack at 4°C for 20 min. Subsequently, they were centrifuged at 4,000 rpm for 10 min within 2 h. Following centrifugation, 200 uL of the supernatant was transferred to individual Eppendorf tubes using a pipette, with 200 uL in each tube. Four tubes were prepared, each labeled with a marker indicating the sample number and grouping information. The plasma samples were then stored in a −80°C freezer.

### Sample pretreatment

Samples were thawed at 4°C for 60 min, then 50 µL were aspirated into an Eppendorf tube and mixed precisely with 300 µL of methanol solution. Subsequently, 1 mL of MTBE was added, and the mixture was vortexed and shaken at room temperature for 30 min. Next, 250 µL of water was added, followed by centrifugation at 15,000 rpm at 4°C for 10 min 100 μL of the supernatant was carefully transferred to a labeled centrifuge tube, dried in a vacuum concentrator, and stored under sealed, low-temperature conditions. Prior to analysis, 200 μL of acetonitrile-isopropanol solution was added to the samples, followed by vortex mixing. The samples were then centrifuged at 15,000 rpm for 10 min at 4°C.

### Sample separation and mass spectrometry

Lipid separation was carried out using the Ultimate 3000 ultra-high-performance liquid chromatography system. The chromatographic system utilized an Accucore C30 core-shell column (2.6 μm, 2.1 mm × 100 mm) operating at a column temperature of 50°C. Mobile phase A consisted of a 6:4 ratio of acetonitrile to water, containing 10 mmol ammonium formate and 0.1% formic acid, while mobile phase B was composed of a 1:9 ratio of acetonitrile to isopropanol, supplemented with 10 mM ammonium formate and 0.1% formic acid. The flow rate for the chromatographic separation was set at 0.3 mL/min. The gradient of the mobile phase was as follows: initial composition of 90%A and 10%B, 50% each of A and B at the fifth minute, and 0%A and 100%B at the 23rd minute maintaining this ratio for column washing and equilibration until the 30th minute.

Lipid mass spectrometry analysis was performed using a Q-Exactive hybrid quadrupole-orbitrap mass spectrometer. Electrospray ionization was employed as the ion source, generating positive and negative ions. For the front ionization conditions, the sheath gas flow rate was set at 45 arb, auxiliary gas flow at 10 arb, ionization chamber heated to 355°C, capillary temperature at 320°C, and S-Lens RF level at 55%. Lipid molecular ions were scanned using a full scan mode at a resolution of 70,000 FWHM, with a maximum injection time of 200 milliseconds and a mass-to-charge ratio scan range of 300–2000 m/z. For secondary mass spectrometry fragment ions, a resolution of 17,500 FWHM and a maximum injection time of 80 milliseconds were utilized. Nitrogen gas was used as the collision gas, with a dynamic exclusion time of 8 s.

### Quality control (QC) analysis

QC samples were utilized for sample mixing. The variability coefficient (CV value) between QC samples was calculated to assess the overall stability of the experiment. Moreover, the correlation between QC samples was calculated to evaluate the experimental reproducibility. Finally, the distances and clustering tightness among different QC samples in the principal component analysis (PCA) score plot were visually shown for intuitive demonstration.

### Data collection

Lipid compositional analysis was conducted using the LipidSearch software (Thermo Scientific, United States) in conjunction with the LipidMaps database. The chromatographic peak areas (AUC) were extracted using the TraceFinder software to provide relative quantitative information.

### Data processing and analysis

Measurement values were expressed as means ± standard deviation (mean ± SD), while count data were represented using ratios. PCA and orthogonal partial least squares discriminant analysis (OPLS-DA) multivariate statistical analysis were performed using SIMCA-P software (Umetrics, Sweden) (Alseekh et al., 2021; Bartel et al., 2013). T-tests, one-way analysis of variance (ANOVA), and false discovery rate (FDR) corrected unit statistical analysis were conducted using SPSS 26.0 software (IBM, United States) (Bar et al., 2020). A diagnostic model was constructed based on binary logistic regression. Random Forest (RF) algorithm and the glmnet package in R version 4.0.3 environment (R Core Team, 2020) were employed to further screen differential lipids, optimizing and simplifying lipid selection using LASSO regression from the glmnet package. MetaboAnalyst was utilized for heatmap visualization and lipid functional enrichment analysis, while the Biopan tool was used for lipid-lipid interaction analysis (Xia and Wishart, 2010). Receiver operating characteristic (ROC) curves and boxplots were generated using GraphPad Prism 9.0 (Gaud et al., 2021).

### Construction of the diagnostic model

This study used Logistic regression, LASSO regression, and receiver operating characteristic (ROC) curve analysis to assess the diagnostic value of metabolite molecules for HLAP. LASSO regression was first employed during the model construction process to select features among the identified lipid molecules. The selection criterion was the mean squared error (MSE) minimized penalty parameter λ, which was determined through 10-fold cross-validation to choose the optimal λ value, thereby reducing redundant variables and preventing overfitting. The selected variables were then input into a Logistic regression model to construct the diagnostic model. The parameters of the Logistic regression model were optimized using the maximum likelihood estimation (MLE) method. The model’s predictive performance was further evaluated through 10-fold cross-validation, ultimately selecting the model parameters with the largest area under the AUC and minimal bias as the optimal parameters. In each cross-validation, the data were randomly divided into a training set (70%) and a testing set (30%) to train and validate the model.

## Ethical statement

This study has been approved by the Ethics Review Committee of the affiliated hospital of North Sichuan Medical College (approval number: 2021ER133-1). Before participating in the study, all individuals were provided with comprehensive information regarding the research objectives, methods, potential risks, and benefits, and after full understanding, they voluntarily signed an informed consent form. The study strictly adhered to relevant medical research ethics standards and international medical ethical guidelines, ensuring the protection of participants’ privacy and data security, with no additional health risks imposed on the participants. All collected samples and clinical information were kept strictly confidential and used solely for this study.

## Results

### Baseline data comparison

This study included 24 HLAP patients (18 with mild severity and 6 with moderate severity) and 24 healthy controls. The baseline data analysis revealed that the white blood cell count and neutrophil percentage in the HLAP group were significantly higher compared to the Control group. Furthermore, the TG and cholesterol levels were markedly elevated in the HLAP group, along with varying degrees of increase in different types of lipoproteins, indicating a significant lipid metabolism disorder occurring in the HLAP group, as presented in Table 1.

**TABLE 1:** Baseline data comparison between HLAP group and con group.

Item	Con (n = 24)	AP (n = 24)	P value
Age (years)	53.47 ± 18.6	54.81 ± 15.29	—
Gender (Male/Female)	14/10	14/10	0.35
White Blood Cell Count (*10^9^/L)	5.62 ± 1.24	10.49 ± 3.54	1
Neutrophil Percentage (%)	57.04 ± 6.6	79.57 ± 10.52	4.53*10^–8^
Hematocrit	38.89 ± 5.36	35.88 ± 14.38	1.30*10^–10^
Creatinine (μmol/L)	61.26 ± 15.54	57.65 ± 21.01	0.18
Urea (μmol/L)	5.9 ± 1.79	4.66 ± 1.69	0.08
Blood Glucose (mmol/L)	5.45 ± 0.86	9.18 ± 3.84	0.11
Alanine Aminotransferase (U/L)	21.88 ± 17.33	125.8 ± 150.96	6.93*10^–6^
Aspartate Aminotransferase (U/L)	22.82 ± 10.29	94 ± 108.14	0.24
Total Bilirubin (μmol/L)	12.48 ± 3.81	29.86 ± 42.05	0.12
Direct Bilirubin (μmol/L)	3.58 ± 1.41	14.4 ± 13.94	0.14
Total Cholesterol (mmol/L)	4.82 ± 0.8	7.47 ± 3.97	0.03
Triglycerides (mmol/L)	1.59 ± 0.75	8.8 ± 8.11	2.31*10^–5^
High-density Lipoprotein (mmol/L)	1.18 ± 0.27	1.53 ± 1.43	3.03*10^–6^
Low-density Lipoprotein (mmol/L)	2.6 ± 0.55	2.65 ± 1.04	0.04

### Metabolomics data quality assessment

**FIGURE 1:**
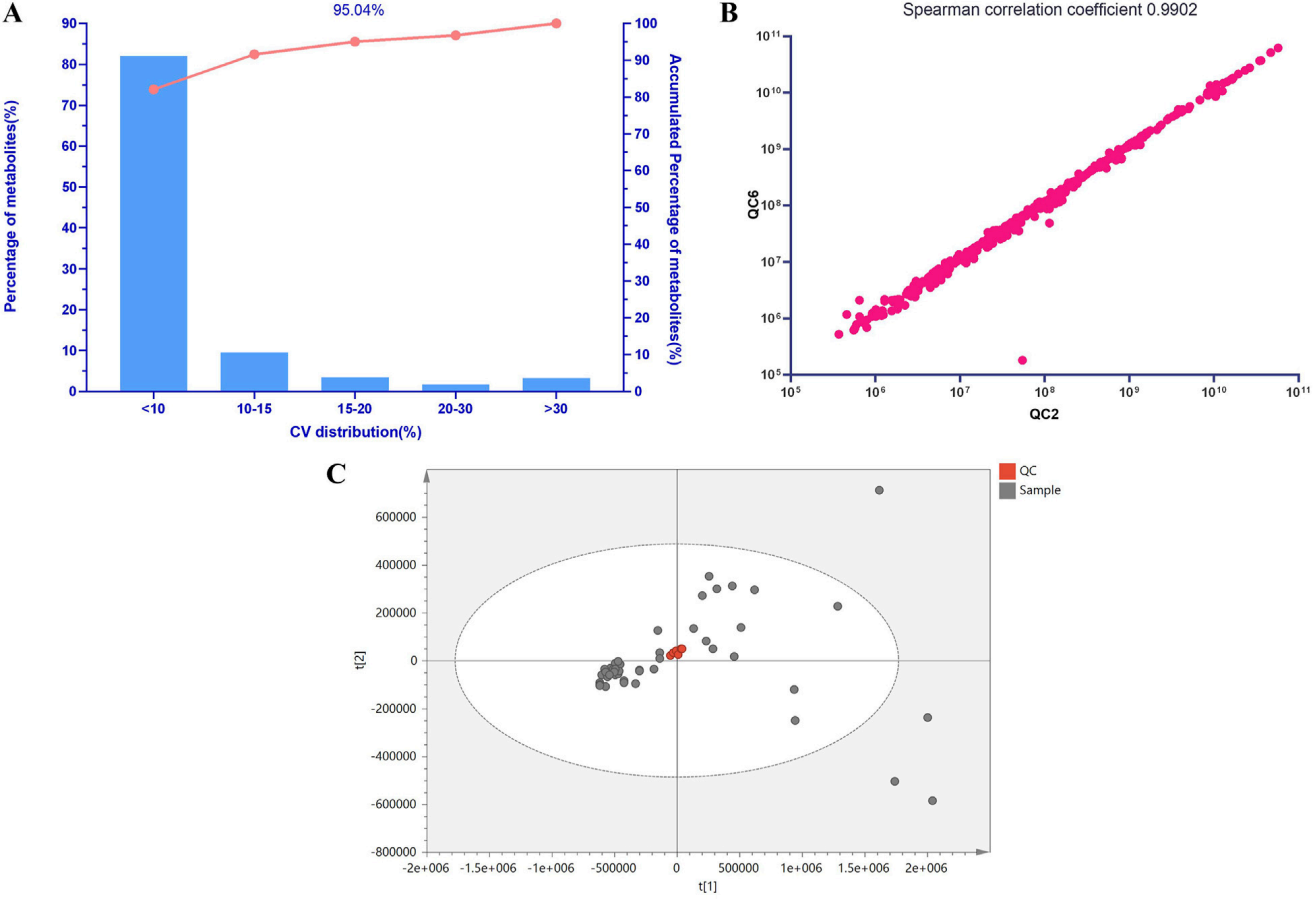
Lipid Molecular QC Sample Analysis. Note: **(A)** Pareto Chart of Coefficients of Variation for Lipid Molecular QC Samples. The *x*-axis represents the percentage range of coefficients of variation in the lipid data of QC samples used for statistical analysis, while the *y*-axis represents the percentage of lipids within this range. The curve in the graph shows the cumulative percentage across different ranges of coefficients of variation. **(B)** Scatter Plot of Spearman Correlation Coefficients for Lipid Molecular QC Samples. **(C)** PCA Score Plot of Lipid Molecular QC Samples.

This study utilized a Pareto chart to visually assess the quality of the metabolomics data results (Figure 1A). It was observed that 95.04% of the lipid relative standard deviation (RSD) values were below 20% based on the Pareto chart. Figure 1B demonstrates that even among all QC samples, selecting the ones with the lowest correlation, their Spearman correlation coefficient remained high at 0.9902. Furthermore, the distribution of QC samples in Figure 1C further confirms the stability and reliability of the lipid results. Given the complexity of the human body, this study utilized 999 permutation tests to evaluate the robustness of the OPLS-DA model. The analysis revealed that within the blue healthy control group, there was minimal variation, and they were notably separated from the red AP group along the first principal component, indicating a significant segregation trend. Consistent with clinical knowledge, the majority of lipid levels in patients with HLAP showed significant differences compared to healthy individuals, while the lipid levels in healthy individuals remained stable and similar to each other.

### Identification of differential metabolites

**FIGURE 2:**
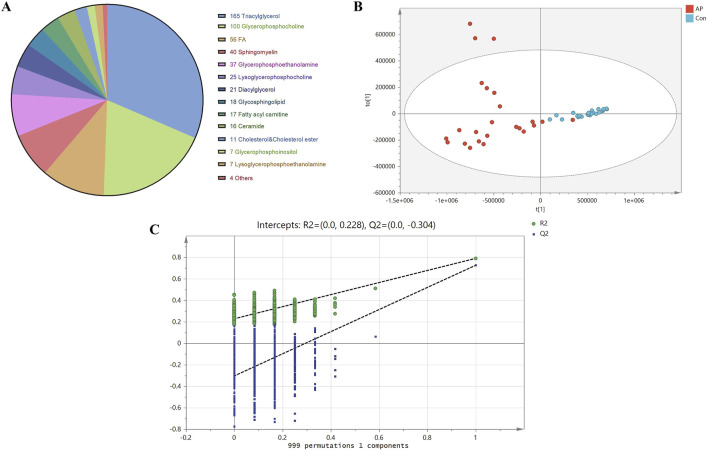
Lipid Molecular Composition, OPLS-DA Scores, and Permutation Test Analysis. Note: **(A)** Lipid molecular composition of different classes. **(B)** OPLS-DA score plot of lipid molecules between HLAP and Con groups. Blue dots represent healthy control samples (Con), while red dots indicate hyperlipidemia pancreatitis samples. **(C)** Permutation test of OPLS-DA model between HLAP and Con groups.

All samples underwent analysis on the LC-MS lipidomics platform. Both univariate and multivariate analysis models were employed following substance identification, data QC, and data transformation. A total of 524 lipid species were identified after screening. TG was the most abundant, followed by phosphatidylcholines (PC), with as many as 56 different types of free FA. The composition of lipid molecules from various classes is illustrated in Figure 2A.

Due to the complexity of the human body, variations within and between the two study groups are often intertwined, making the use of PCA alone insufficient to achieve clear and meaningful inter-group classification results. Therefore, OPLS-DA was utilized to maximize the classification of lipids between groups. To avoid overfitting due to excessive classification, we employed a permutation validation approach, which involved iteratively rearranging the sample classification in the original dataset to create new models and estimate their parameters. This method is known as permutation validation. The robustness of the OPLS-DA model was evaluated in this study through 999 permutations, as shown in Figure 2B.

The results of the 999 permutation validations shown in Figure 2C indicate that the intercept of the model prediction ability Q2 was −0.304, and the intercept of the model explanatory power R2 was −0.228. The positive slopes of both intercepts indicate a robust model without overfitting.

To balance the false negative and false positive rates, we applied the FDR correction to the p-values of the t-test using the Benjamini-Hochberg method. A threshold of q < 0.05 led to the initial identification of 393 differentially expressed lipids.

### Identification of differential metabolites

**FIGURE 3:**
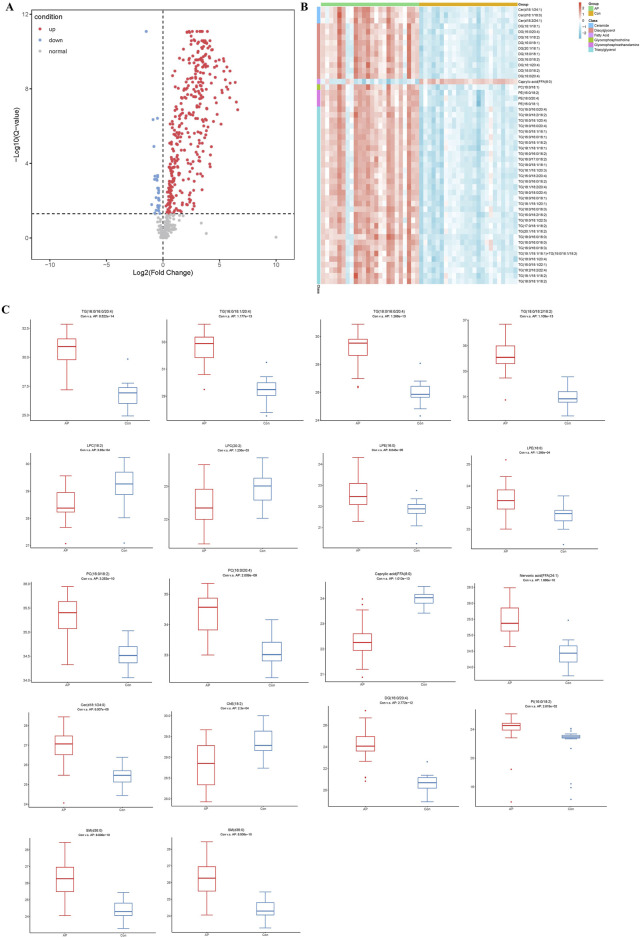
Analysis of Differential Lipid Molecules. Note: **(A)** Volcano plot of lipid molecules between HLAP and Con groups. **(B)** Heatmap of the relative abundance of differential lipid molecules. **(C)** Box plot of various types of differential lipid molecules between HLAP and Con groups. The *y*-axis represents the relative abundance of lipid molecules compared to the control group. TG: Triglycerides; LPC: Lysophosphatidylcholine; PC: Phosphatidylcholine; Caprylic acid: Octanoic acid; Nervonic acid: Nervonic acid; Cer: Ceramide; ChE: Cholinesterase; DG: Diglycerides; PI: Phosphatidylinositol.

The volcano plot in Figure 3A visually represents the general lipid distribution between the two groups. In the pathological state of HLAP, the average abundance of most lipids is higher than in the healthy population. To visually assess the relative concentrations of each specific lipid in the samples, a heatmap corresponding to lipids with significant differences in the volcano plot was generated. As depicted in Figure 3B, the specimens from the AP and Con groups are divided into two groups from left to right. These distinct lipid categories include ceramides (Cer), diacylglycerols (DG), FFA, PC, phosphatidylethanolamines (PE), and predominantly TG. Subsequently, box plots (Figure 3C) were created to intuitively explore the various classes of differential lipids. The most abundant lipid category between the two groups is TG, with the majority of TG levels elevated in the AP group, aligning with clinical observations and definitions. The fatty acid chains in TG contain the common inflammation factor arachidonic acid (20:4). Differential lysophosphatidylcholines (LPC) decrease in concentration during hyperlipidemic pancreatitis, whereas lysophosphatidylethanolamines (LPE) show increased concentrations during this condition. PC, the precursors of LPC, exhibit the opposite trend, with concentrations increasing during hyperlipidemic pancreatitis, suggesting a conversion between PC and LPC. The relative concentrations of free FA show diverse variations, with shorter saturated FA and sphingolipids having lower concentrations in the diseased state, while long-chain unsaturated free FA and sphingolipids have higher concentrations in the diseased state. Furthermore, the relative expression intensity of various representative lipid categories is depicted in the figure.

### Systems biology analysis of the impact of hyperlipidemia on lipid metabolism pathways

In this study, we utilized systems biology tools to investigate the specific effects of hyperlipidemia on human metabolic pathways. By integrating the KEGG database and Biopan tool, we conducted an in-depth analysis of metabolic differences between healthy individuals and patients with hyperlipidemia.

**FIGURE 4:**
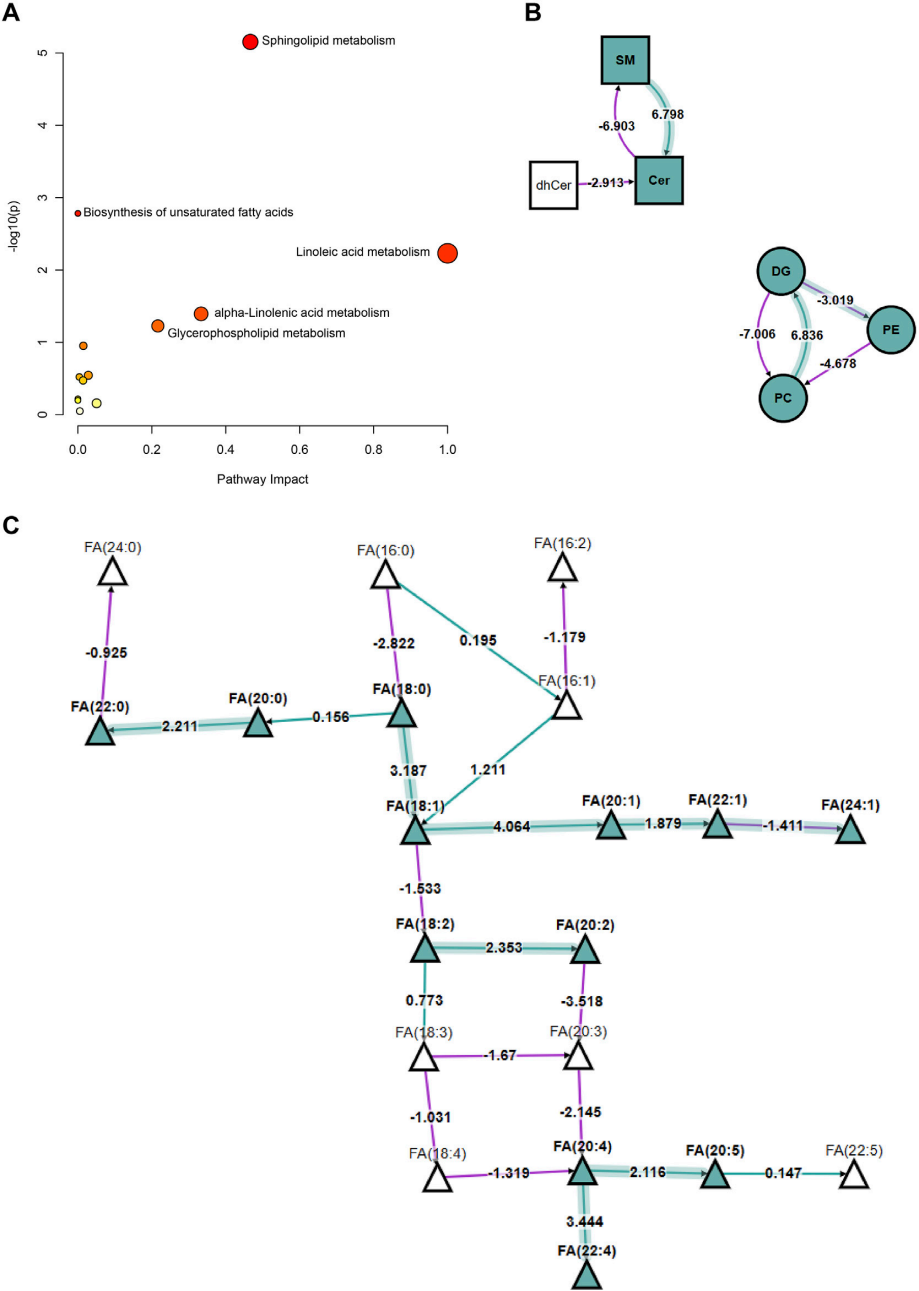
Analysis of Changes in Lipid Metabolism Pathways in HLAP and Control Groups. Note: **(A)** Represents the significant differences and pathway impact values in various metabolic pathways between the hyperlipidemia and healthy control groups. The significance level is indicated by −log (p) values, and the pathway impact values are calculated based on the degree of metabolite changes. Each circle in the figure represents a specific metabolic pathway, with the size of the circle reflecting the significance of metabolite changes at key nodes within the pathway, and the color depth indicating the importance of the pathway in the analysis. This analysis is based on the KEGG database’s metabolic data from 57 hyperlipidemia patients and healthy controls. **(B)** Describes the interconversion relationships among lipid compounds, including SM, Cer, DG, PE, PC, and other major lipid classes dynamic changes. In the figure, arrows and numbers denote the regulatory direction and strength of the corresponding pathways, with transformation pathways analyzed using the Biopan tool. **(C)** Displays the expression differences of specific fatty acid species between the hyperlipidemia and healthy control groups. Triangles in the figure represent different types of FA, with the color and direction of arrows indicating the trend of concentration increase or decrease. The data is derived from metabolite profiling of 57 study subjects. Statistical methods employed in the figure include t-tests, with a significance level set at *p* < 0.05. Abbreviations such as “SM” and “Cer” specially marked in the figure represent sphingomyelin and cerebrosides, aiding in the rapid identification of metabolic changes in relevant lipid classes.

Figure 4A illustrates the relationship between various metabolic pathways’ significance levels (−log (p) values) and their pathway impact values. Pathways such as sphingolipid metabolism, unsaturated fatty acid biosynthesis, linoleic acid metabolism, α-linolenic acid metabolism, and glycerophospholipid metabolism showed significant differences, indicating their substantial regulation under hyperlipidemic conditions. Figure 4B provides a detailed explanation of the interconversion of lipids. In hyperlipidemia, the conversion process from SM to Cer is enhanced, while pathways from DG to PE and PE to PC are upregulated; conversely, the conversion from PC to DG is downregulated. These findings suggest hyperlipidemia significantly influences key lipid conversion pathways essential for cell membrane structure and function. Figure 4C further reveals the expression changes of specific FA in hyperlipidemia, such as a significant increase in the concentration of FA (18:1), which may have profound effects on cellular metabolic functions. In conclusion, our study conclusively demonstrates the broad impact of hyperlipidemia on human lipid metabolism pathways and reveals its potential biochemical mechanisms.

### Building HLAP prediction models

**FIGURE 5:**
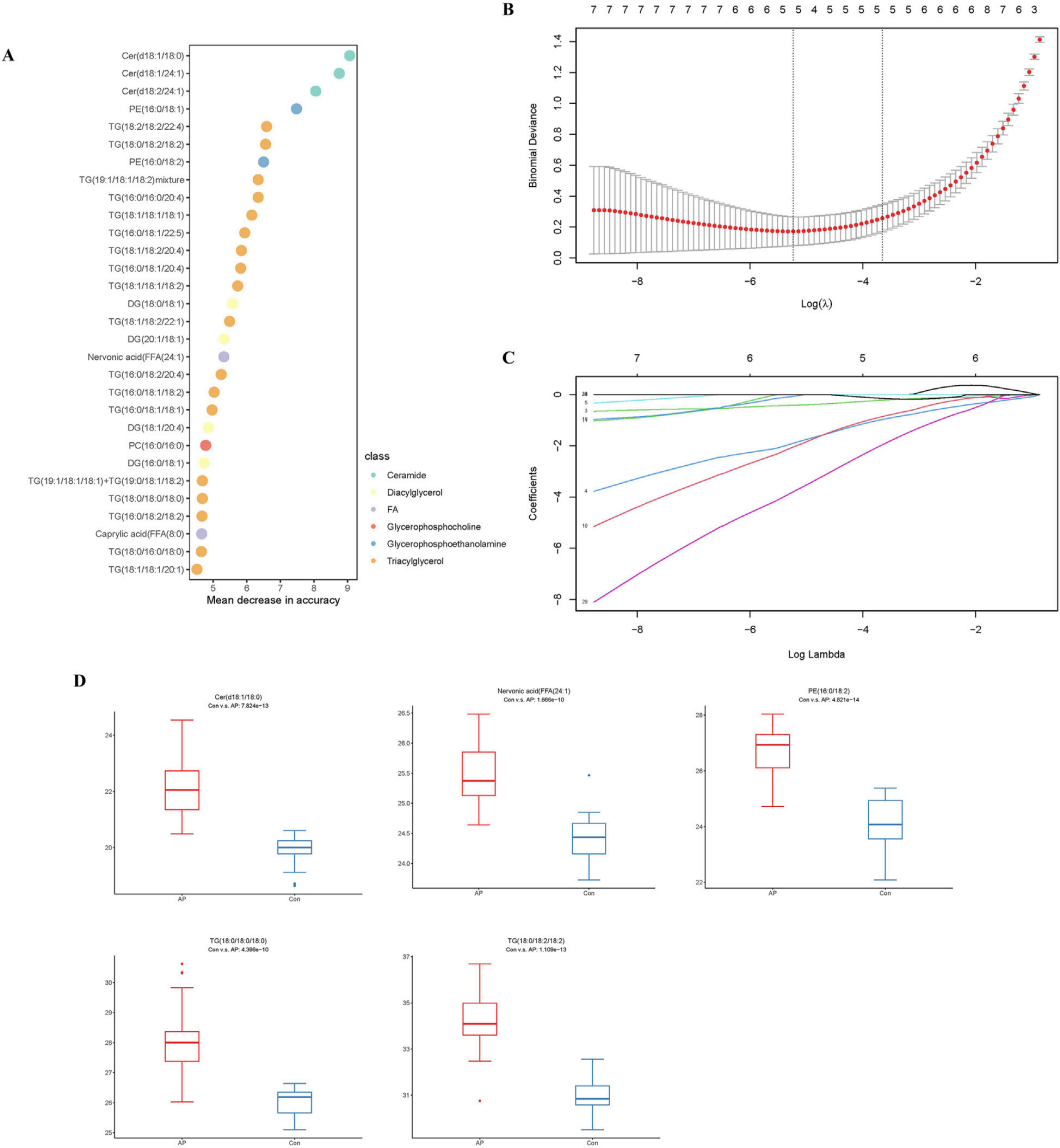
Analysis of the HLAP Prediction Model. Note: **(A)** RF analysis. **(B)** Selection of the optimal lambda threshold. **(C)** Determination of the metabolite coefficient. **(D)** Box plots of the expression of candidate biomarkers.

To further refine the 393 differentially expressed lipids, we utilized the RF package in the R environment for weighting these lipids and ranking the top 30 lipids (Table 2) based on their compound classification, represented by a bubble plot in Figure 5A. Subsequently, a LASSO regression was performed on these 30 lipid classes (Figures 5B, C), resulting in the identification of the top five lipids under the optimal threshold: TG (18:0/18:2/18:2), PE (16:0/18:2), Cer(d18:1/18:0), Nervonic acid, and TG (18:0/18:0/18:0), all showing higher concentrations in hyperlipidemic pancreatitis as depicted in the box plots (Figure 5D).

**TABLE 2:** Lipid molecules with significant differences between HLAP and con groups.

Biomarker	Formula	m/z	p-value	q-value	Log2 (AP/Con)
Caprylic acid	C8H16O2	143.1078	1.01343E-13	8.30251E-12	−1.485470961
TG (16:0/16:0/20:4)	C55H98O6	872.77017	8.52228E-14	8.30251E-12	3.926289986
TG (18:0/18:2/18:2)	C57H102O6	900.80147	1.10935E-13	8.30251E-12	3.476823726
PE (16:0/18:2)	C39H74NO8P	716.52248	4.82072E-14	8.30251E-12	2.594308018
TG (16:0/18:1/20:4)	C57H100O6	898.78582	1.17709E-13	8.30251E-12	3.261769776
TG (16:0/18:1/18:1)	C55H102O6	876.80147	1.20791E-13	8.30251E-12	2.304980798
TG (16:0/18:1/18:2)	C55H100O6	874.78582	1.53237E-13	8.92182E-12	2.124124598
Cer(d18:1/24:1)	C42H81NO3	648.62892	1.95157E-13	9.29656E-12	2.127801342
TG (18:1/18:1/18:1)	C57H104O6	902.81712	3.38151E-13	1.47659E-11	2.489301105
Cer(d18:1/18:0)	C36H71NO3	566.55067	7.8236E-13	2.6129E-11	2.50696111
PE (16:0/18:1)	C39H76NO8P	718.53813	9.01743E-13	2.62507E-11	2.813541801
TG (16:0/18:2/20:4)	C57H98O6	896.77017	9.52203E-13	2.62608E-11	3.441453685
TG (18:1/18:2/20:4)	C59H100O6	922.78582	1.29421E-12	3.08258E-11	3.144735622
TG (18:1/18:1/20:1)	C59H108O6	930.84842	1.77608E-12	3.66702E-11	3.868022765
TG (16:0/18:2/18:2)	C55H98O6	872.77017	2.11001E-12	4.09497E-11	2.474100221
TG (16:0/18:1/22:5)	C59H102O6	924.80147	3.45723E-12	6.03864E-11	3.421334486
DG (16:0/18:1)	C37H70O5	612.55615	3.79454E-12	6.414E-11	3.976919034
Cer(d18:2/24:1)	C42H79NO3	646.61327	4.65157E-12	7.38614E-11	1.812479291
DG (20:1/18:1)	C41H76O5	666.6031	5.09199E-12	7.84766E-11	4.36872368
DG (18:0/18:1)	C39H74O5	640.58745	5.46938E-12	8.18844E-11	4.2989165
TG (18:0/16:0/18:0)	C55H106O6	880.83277	7.84752E-12	1.11138E-10	3.08317512
DG (18:1/20:4)	C41H70O5	660.55615	1.203E-11	1.61634E-10	3.72486085
TG (19:1/18:1/18:1)	C58H106O6	916.83277	1.82442E-11	2.22325E-10	4.126769444
TG (18:2/18:2/22:4)	C61H102O6	948.80147	2.40249E-11	2.62271E-10	3.231330656
TG (18:1/18:1/18:2)	C57H102O6	900.80147	2.54852E-11	2.72535E-10	2.283815096
PC(16:0/16:0)	C40H80NO8P	734.56943	2.94853E-11	2.79872E-10	1.790429473
TG (19:1/18:1/18:2)	C58H104O6	914.81712	3.23476E-11	2.97371E-10	3.412513352
TG (18:1/18:2/22:1)	C61H110O6	956.86407	3.60066E-11	3.25301E-10	5.385513944
Nervonic acid	C24H46O2	365.3425	1.66645E-10	1.10534E-09	1.054612444
TG (18:0/18:0/18:0)	C57H110O6	908.86407	4.39593E-10	2.55941E-09	2.579675504

**FIGURE 6:**
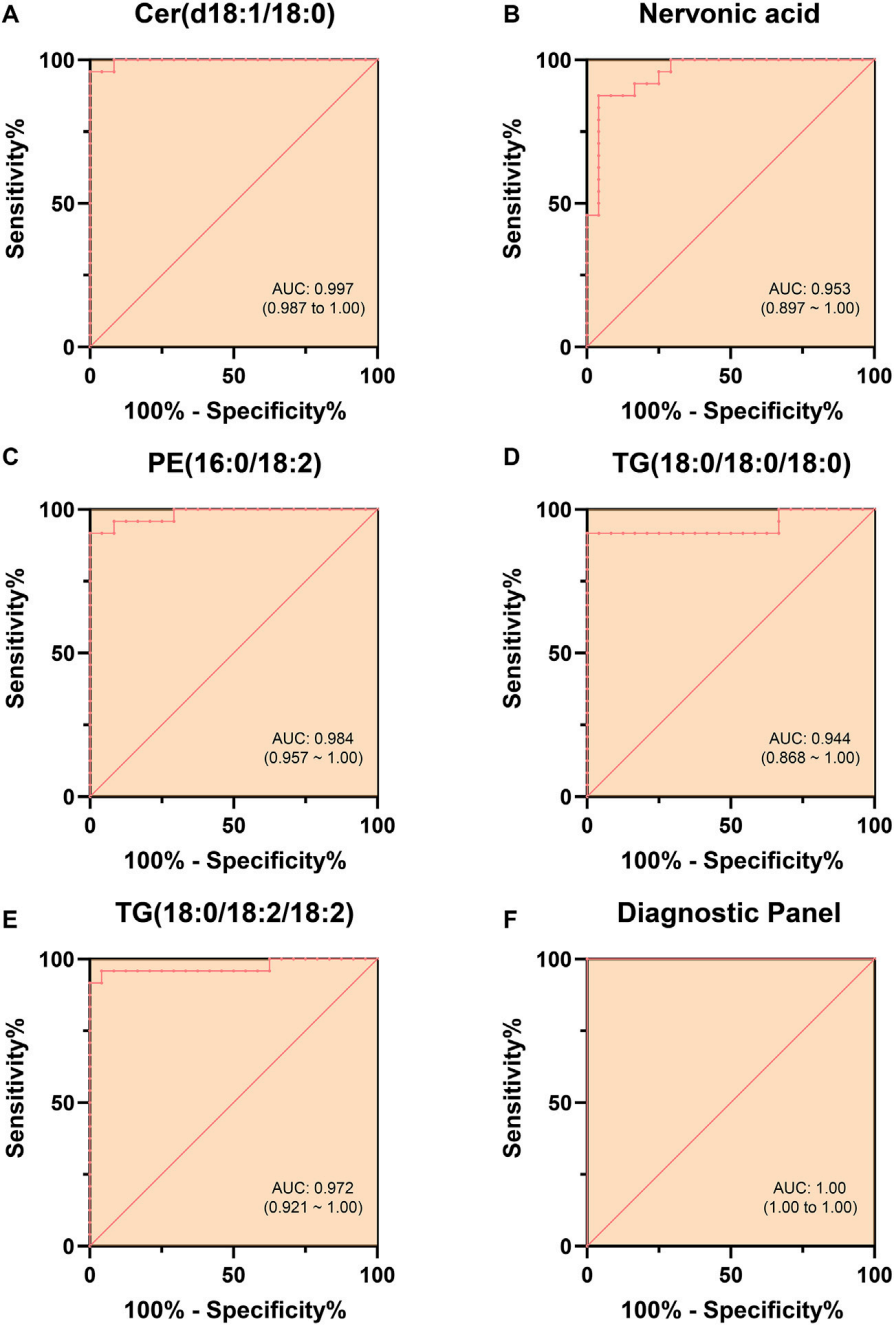
ROC Curves Differentiating HLAP from Con Using Individual and Combined Models. Note: **(A–E)** depict the ROC curves for five lipid molecules (Cer(d18:1/18:0), sphingosine, PE (16:0/18:2), TG (18:0/18:0/18:0), and TG (18:0/18:2/18:2)) in the diagnosis of hyperlipidemia. Each ROC curve in the figures assesses the diagnostic performance of the respective lipid molecule, with the range of the AUC provided in the graph, offering quantitative indicators of each lipid molecule’s diagnostic sensitivity and specificity. **(F)** Combining the ROC curves of these five lipid molecules forming the diagnostic panel showcases the performance of the composite diagnostic index. The statistical significance level was set at *p* < 0.05. The term “AUC” in the figures represents the English abbreviation for “area under the curve,” indicating the diagnostic accuracy of the model.

ROC curves were generated (Figures 6A–E) to evaluate the diagnostic performance of these five lipid molecules individually. Each of these molecules exhibits good sensitivity and specificity as standalone indicators (Table 3). Moreover, when these five lipid molecules are combined into a logistic regression model, the diagnostic model achieves an AUC 1, indicating a complete differentiation between hyperlipidemic pancreatitis and healthy individuals (Figure 6F).

**TABLE 3:** Characteristics of differential changes in candidate biomarkers and AUC values.

Biomarker	Sensitivity%	Specificity%	AUC
TG (18:0/18:2/18:2)	91.7	100	0.972
PE (16:0/18:2)	91.7	100	0.984
Cer(d18:1/18:0)	95.8	100	0.997
Nervonic acid	87.5	95.8	0.953
TG (18:0/18:0/18:0)	91.7	100	0.944
Diagnostic Panel	100	100	1

## Discussion

The pancreas is a vital metabolic organ that regulates the body’s metabolic homeostasis (Saltiel and Kahn, 2001). The pathophysiological mechanisms of general AP primarily involve abnormal activation of zymogens within the pancreas, pancreatic cell injury, and the cascade effects of the inflammatory response (Banks et al., 2006). Metabolic disturbances are one of the hallmark features of acute pancreatitis. Compared to general AP, HLAP patients not only experience the common pathological processes of AP but also exhibit significant lipid metabolism disorders, making the pathophysiological mechanisms of HLAP more complex and diverse. In hyperlipidemia, plasma TG levels are significantly elevated, and under the action of pancreatic lipase, they are hydrolyzed into large amounts of FFA. The accumulation of FFAs not only directly damages pancreatic cells but also exacerbates pancreatic injury by inducing oxidative stress and inflammatory responses (Guo et al., 2019).

In this study, the serum lipid profiles of 24 HLAP patients and 24 healthy controls were analyzed utilizing the LC-MS Lipidomics platform. Differential analysis revealed significant alterations in TG, FFA, glycerophospholipids, and sphingolipids in the HLAP group. Furthermore, this study’s comprehensive description of the lipid metabolic features in HLAP patients holds significant scientific value, laying the groundwork for future diagnostic and therapeutic strategies. Clinically, these findings enhance the management and prognosis assessment of HLAP. Additionally, the specific pathways and molecular mechanisms of lipid metabolism mentioned in this study provide potential targets for drug development and therapeutic interventions against HLAP.

Insufficient understanding of the pathogenesis of HLAP has long been a primary reason for the lack of specific therapies for this disease. Due to limitations in detection throughput, previous research on HLAP has primarily focused on TG and FFAs (Yang and McNabb-Baltar, 2020; Kuklinski et al., 1991). Elevated TG and FFAs in the blood are independent risk factors for AP accompanied by organ dysfunction (Kuklinski et al., 1991). This study not only confirmed the significant elevation of TG levels in HLAP patients but also identified varying concentrations of different TG subtypes in these patients, which may explain why only a portion of high TG patients progress to HLAP. Guidelines recommend plasma exchange or insulin administration to increase lipoprotein lipase activity for reducing TG levels (Zou et al., 2005). However, treatments to lower blood lipids have not significantly reduced complication rates and mortality among patients. The diverse nature of lipids, being insoluble in water and soluble in organic solvents, presents a challenge. It is speculated that the lack of improvement in the pro-inflammatory lipid composition due to indiscriminate plasma lipid replacement may account for poor treatment outcomes. Through in-depth research on the plasma lipid profile of HLAP, replacing a specific lipid type or regulating certain specific lipid metabolism pathways may offer promising applications.

Liu et al. (2024) conducted a comprehensive metabolomics and lipidomics analysis, revealing significant changes in TG, PC, and PE in the serum of AP patients. The study highlighted that the levels of these lipid molecules significantly alter with increasing severity of AP, suggesting their potential roles in the pathophysiology of the disease. Similar to Liu et al.’s findings, our study also identified significant changes in TG, PC, and PE levels in HLAP patients. Furthermore, through lipidomics analysis, we identified the roles of additional lipid molecules, such as nervonic acid and Cer, in HLAP. Although Liu et al.'s research emphasized the association between changes in TG, PC, and PE and the severity of AP, it did not specifically address these alterations in the context of HLAP. Compared to general AP, HLAP presents more pronounced lipid metabolism abnormalities. The findings from Liu et al.'s 2024 study complement our results, highlighting the importance of lipid metabolism in acute pancreatitis.

Recent research has confirmed that high concentrations of long-chain unsaturated FA are the main cause of pancreatic acinar cell damage leading to HLAP. In this study, we observed an increase in FA concentration, with enriched analysis of lipid molecular pathways revealing enhanced synthesis of long-chain unsaturated FA. The presence of TG molecules with an 18-carbon fatty acid chain notably increased in HLAP, suggesting that these TG subtypes may serve as the material basis for FFA metabolism in HLAP. Modulating the synthetic enzymes involved in FFA metabolism could be a potential therapeutic target for HLAP. LPC and LPE are common bioactive lipids in serum, serving as key metabolic products in cells and are involved in membrane construction and signal transduction (Trinder et al., 2019; Yang et al., 2019). PC and PE are hydrolyzed by phospholipase A2 (PLA2) at the sn-2 position of the phospholipid, generating LPC and LPE. Studies have indicated that LPC exhibits pro-inflammatory activity and can promote cell apoptosis. Biopan is a cutting-edge tool for lipid metabolic pathway analysis (Deng et al., 2021; Costello, 2018). Analysis based on the Biopan database revealed an increase in the conversion of DG to PE and PC. TG hydrolysis yields DG after releasing a fatty acid chain. Elevated concentrations of PE and PC lead to the formation of LPC and LPE under the action of phospholipase, exerting pro-inflammatory effects. Additionally, PE and PC serve as precursor molecules for leukotrienes, prostaglandins, and other inflammatory mediators. The shift of lipid molecular, metabolic subtypes in HLAP towards the conversion into pro-inflammatory mediators provides a substantial material foundation for the inflammatory mediators required in systemic inflammatory responses.

Based on five lipid molecules, the diagnostic model constructed in this study demonstrated predictive solid performance, with an area under the AUC of 1, indicating its potential clinical application in distinguishing HLAP. For instance, this diagnostic model could be used for the early identification of high-risk patients, enabling more timely and personalized interventions. Additionally, the lipid molecules included in the diagnostic model could be utilized to monitor disease progression and prognosis. Continuous monitoring of changes in these lipid molecules during treatment may assist physicians in evaluating therapeutic efficacy and making necessary adjustments to the treatment plan.

However, despite the model’s high efficiency, the limitations of this study, such as the small sample size and the constraints of the study design, necessitate further validation of our findings in larger cohorts. Moreover, the high cost of lipidomics analysis and the need for specialized technical expertise may limit the widespread clinical application of this diagnostic model. Therefore, simplifying the analysis process, reducing costs, and enhancing the practicality of the results are crucial challenges for the broader adoption of this model in clinical practice.

Additionally, due to the use of a non-targeted lipidomics platform, while it can describe a wide range of lipid molecules, the exploration depth of specific pathways is limited, and some conclusions will need further validation through targeted lipidomics techniques in the future. Lastly, as this study is cross-sectional, the causal relationship between lipid molecules and disease states remains unclear and requires further investigation in larger sample sizes and prospective studies. Therefore, future research should focus on the early diagnosis of diseases and a detailed analysis of the pathological mechanisms, as well as the specific roles and mechanisms of these lipid metabolic changes in disease progression, especially how they impact the severity of pancreatitis and treatment response. Additionally, our study emphasizes the importance of utilizing a variety of bioinformatics tools and databases, such as Biopan and KEGG, for a detailed analysis of lipid metabolic pathways. These tools helped us uncover the comprehensive picture of lipid metabolism in the HLAP pathological state, including changes in key metabolites and metabolic pathways. This approach provides a robust framework for future lipidomic studies to more comprehensively understand complex diseases’ metabolic networks and develop new diagnostic and treatment strategies for clinical applications.

## Conclusion

**FIGURE 7:**
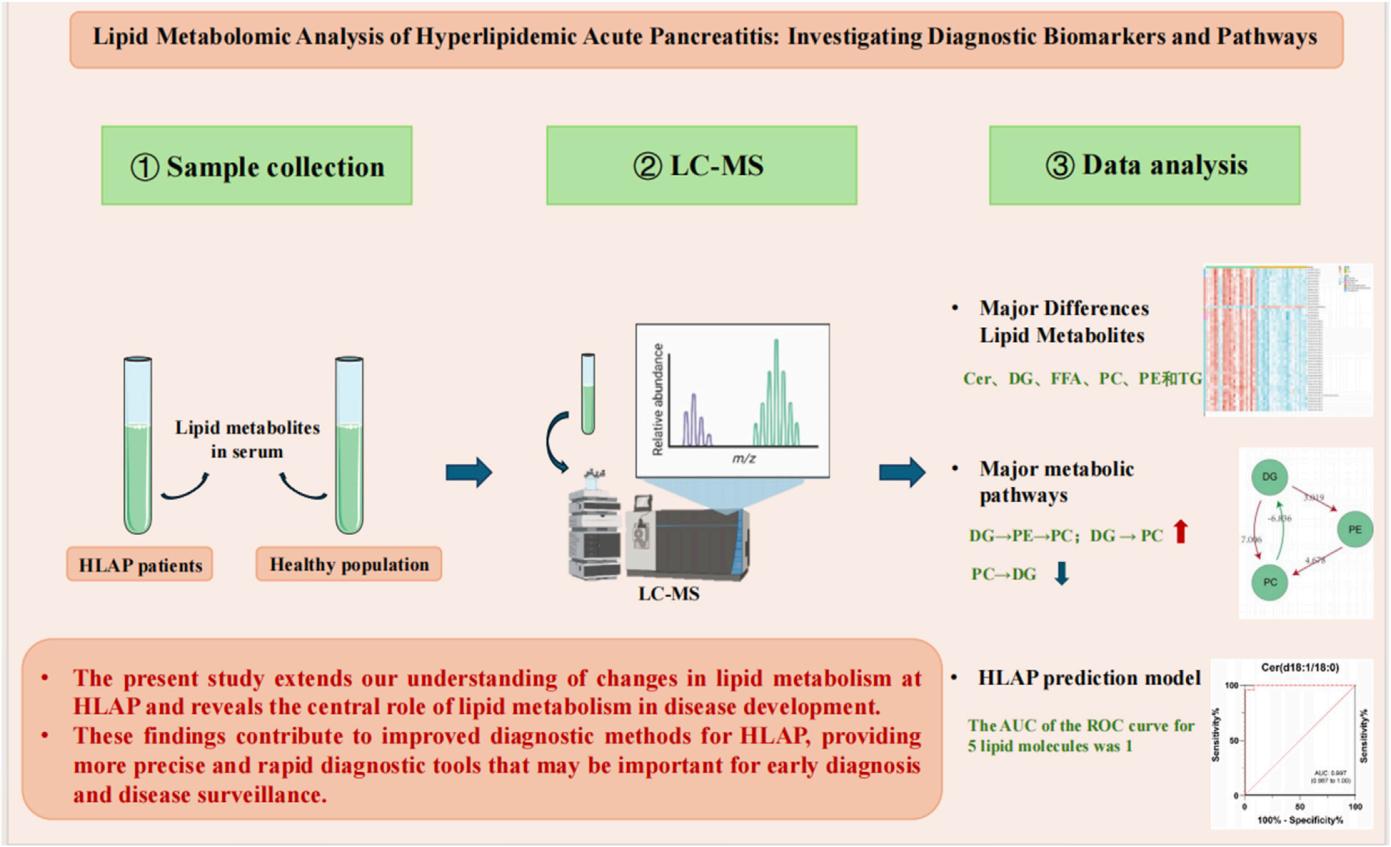
Lipidomics analysis of HLAP: Investigating diagnostic biomarkers and pathways.

This study utilized ultra-high-performance liquid chromatography-tandem mass spectrometry to analyze lipid metabolites in the serum of patients with HLAP compared to a healthy control group, revealing 393 significantly different lipid metabolites between the two groups (Figure 7). Particularly notable were differences in TG, PC, and free FA, involving key metabolic pathways such as sphingolipid, fatty acid, and glycerophospholipid metabolism. Furthermore, a diagnostic model was constructed, utilizing five key lipid molecules, which achieved a highly efficient diagnosis of HLAP, with an area under the ROC curve of 1 indicating exceptional diagnostic accuracy.

Scientifically, this research enhances our understanding of lipid metabolism changes in HLAP and illuminates the central role of lipid metabolism in disease development. This study identifies disease-relevant metabolic markers and demonstrates metabolomics’ potential application in precision medicine. Clinically, these findings contribute to improving diagnostic methods for HLAP, offering more precise and rapid diagnostic tools that could be crucial for early diagnosis and disease monitoring.

Despite the research’s significant scientific and clinical value, some limitations exist. Firstly, the relatively small sample size (24 individuals per group) may restrict the generality and extrapolation of statistical results. Secondly, the study mainly focuses on changes in lipid metabolites, potentially overlooking the role of biomarkers associated with other metabolites. Additionally, as a single-center study, there may be regional biases present.

Future research should aim to increase sample size and engage in multi-center collaborations to validate and optimize the accuracy and applicability of diagnostic models. Furthermore, exploring other types of metabolites, such as amino acids and nucleotides, to comprehensively assess the role of metabolic networks in HLAP is recommended. Moreover, investigating how these metabolites are linked to the clinical manifestations and treatment responses of HLAP will provide valuable insights for developing personalized treatment strategies. Ultimately, these research findings hold promise for translating into new therapeutic targets, offering novel directions for managing HLAP.

## Data Availability

The original contributions presented in the study are included in the article/supplementary material, further inquiries can be directed to the corresponding author.
